# Is Stroke risk analysis (SRA) a reliable method for predicting atrial fibrillation? A systematic review

**DOI:** 10.1371/journal.pone.0305339

**Published:** 2024-06-25

**Authors:** Rafael Alessandro Ferreira Gomes, Michel Pompeu Barros de Oliveira Sá, Marcela Vasconcelos Montenegro, Ludmila Cristina Camilo Furtado, José Henrique Cardoso Ferreira da Costa, Daniel Barros Coutinho, Johnnes Henrique Vieira Silva, Dário Celestino Sobral Filho

**Affiliations:** University of Pernambuco, Recife, Pernambuco, Brazil; Baruch Padeh Medical Center Poriya, ISRAEL

## Abstract

**Introduction:**

Atrial fibrillation is responsible for a considerable number of cases of cardioembolism, accounting for 17% to 30% of the etiologies of all strokes. The software known as Stroke Risk Analysis (SRA) detects patients at high risk of paroxysmal atrial fibrillation by analyzing a continuous electrocardiogram recorded over different periods of time.

**Objectives:**

This article aims to carry out a systematic review investigating the effectiveness of the SRA method in predicting the risk of stroke patients having paroxysmal atrial fibrillation as the cause of the event.

**Methods:**

The methods correspond to the format of the International Prospective Register of Systematic Reviews Protocol, according to CRD Identification Code: CRD42021253974. A systematic search was carried out in BMJB, PubMed/MEDLINE, Science Direct and LILACS. Six cohort studies met the inclusion criteria, representing a total of 2,088 participants with stroke, and compared the detection of patients with paroxysmal atrial fibrillation on the continuous recording electrocardiogram with a time variation of 1 to 48h with the use of SRA.

**Results:**

Studies have shown that SRA has a high negative predictive value (between 96 and 99.1%) and can contribute to the selection of patients at high risk of paroxysmal atrial fibrillation to be referred for implantable cardiac monitoring to continue the investigation.

**Conclusions:**

A sequential combination of SRA with implantable cardiac monitoring is a promising strategy for detecting undiagnosed paroxysmal atrial fibrillation. Thus, the SRA can act as a cost-effective pre-selection tool to identify patients at higher risk of having paroxysmal atrial fibrillation as a possible cause of stroke and who may benefit from implantable cardiac monitoring. However, the lack of randomized studies is a limitation that must be considered.

## Introduction

### ● Justification

Cardioembolism is responsible for 17% to 30% of all ischemic strokes, and some data suggest that more than 50% of these are caused by atrial fibrillation (AF) [1]. It is clear that early detection and treatment of AF are essential, as adequate anticoagulant therapy can reduce the risk of ischemic stroke in patients with this arrhythmia by more than 70% [2].

Secondary prevention of ischemic stroke by detecting patients with atrial fibrillation after ischemic stroke or transient ischemic attack (TIA) is an appropriate strategy, particularly among patients with embolic stroke of unknown origin, as approximately 30% of these patients may have paroxysmal atrial fibrillation (PAF) [3, 4].

AF has a prevalence of 1–2% in the general population. Its paroxysmal form is characterized by successive manifestations, but it disappears spontaneously, usually in less than 48 hours, although it can last for up to 7 days [5]. According to a British study, PAF has an incidence of approximately 1 in 1,000 individuals per year, considering the 40–89 age group [6]. In terms of prevalence, PAF accounts for 25–62% of cases of AF [7]. When studying the interrelationship between paroxysmal AF and the risk of stroke, a recent study of 891 patients with pacemakers had their heart rhythms monitored for 120 days, which showed that the risk of stroke was higher in the first five days after the onset of AF and that the duration of episodes of this arrhythmia, when longer than 5.5 hours, was also associated with higher rates of stroke [8].

Considering that many patients are asymptomatic, the diagnosis of PAF is often challenging, as it may not be detected on conventional electrocardiogram (ECG) and Holter ECG, or well on short-term continuous cardiac monitoring (CCM) [9]. Current guidelines recommend the use of CCM for at least the first 24–72 hours after stroke; however, there is currently no consensus on the optimal method and duration of CCM in stroke unit settings [10].

In modern times, technology has brought numerous advances in the field of medicine. In recent years, a telemedicine technology called Stroke Risk Analysis (SRA), from the manufacturer Apoplex Medical Technologies®, Pirmasens, Germany, has been introduced into medical practice. This analyzes a 1-hour section of a continuous 24-hour electrocardiogram (Holter-24-hour) from a 2-channel ECG to identify patients at high risk of paroxysmal atrial fibrillation, even if the ECG analysis does not include manifestations of AF [11].

The algorithm was developed based on hundreds of datasets of patients with PAF and individuals with no history of AF (validated by cardiologists) [12]. SRA assesses the risk of AF by analyzing the RR interval, forming various mathematical patterns, mainly non-linear, such as deviation of the minor and major axes of the Poincaré graph and the relationship between them; RR fluctuation based on different analyses of consecutive RR intervals; the number of premature atrial complexes without re-establishment in the sinus node; and approximate entropy of the RR interval data (a measure of complexity in analyzing the time series of electrocardiogram data) [13]. The application of new monitoring technologies in the assessment of risk in cardiovascular diseases and their complications is still recent in the literature and there is still no standardization of the methods used. Other systematic reviews have also studied the subject and, in general, conclude that the results achieved are still insufficient for clinical decision-making regarding the validity of SRA for secondary stroke prevention, but do not exclude the possibility that the technique has potential in detecting atrial fibrillation and, consequently, in preventing new ischemic events [1, 14, 15].

### ● Objectives

This article aims to carry out a systematic review investigating the value of SRA in predicting the risk of stroke patients having paroxysmal atrial fibrillation as a cause. The PRISMA (Preferred Reporting Items for Systematic Reviews and Meta-Analyses) protocol was followed [16]. Lastly, this objective was achieved after a systematic analysis of the included studies.

## Methods

### 1) Protocol and registers

The methods in question correspond to the format of a Protocol based on PROSPERO—International Prospective Register of Systematic Reviews—affiliated to the University of York, according to the CRD Identification Code: CRD42021253974. This study did not carry out a meta-analysis due heterogeneous studies.

### 2) Eligibility criteria

The question asked in this review was: What is the role of the SRA in predicting the risk of stroke patients having paroxysmal atrial fibrillation? Based on the PICOS strategy (P: population; I: intervention; C:comparison; O: outcome), studies were considered eligible if: 1—the population was made up of stroke patients; 2—they statistically evaluated the accuracy of the SRA method for detecting PAF; 3—they compared the detection of PAF patients by continuous electrocardiogram with the use of SRA; 4—the population was admitted to a stroke unit after a stroke episode; and 5—they were clinical and/or observational studies [17].

### 3) Sources of information

The following databases were consulted until March 2023: Cochrane Library, British Medical Journal (BMJ) Best Practice, National Library of Medicine National Institutes of Health (PubMed/MEDLINE), Science Direct and Latin American and Caribbean Health Sciences Literature (LILACS). As for the gray literature, it was searched in the Google Scholar and ResearchGate databases. This material was also searched in the references of the scientific articles that were included in the systematic review and in the repositories of course completion papers not yet published in the literature.

### 4) Search strategy

The search was based on the use of controlled vocabulary according to Medical Subject Headings (MeSH). Using the Boolean operators "and" and "or". The descriptors used for this study were: stroke, atrial fibrillation and stroke risk analysis. The search strategy applied to the databases, used the following combination of descriptors: "Atrial Fibrillation"[MeSH] AND “Stroke”[MeSH] AND “Stroke risk analysis” [MeSH].

### 5) Selection process

The search for studies was guided by combinations of descriptors in the databases and the research question. There were no period or language restrictions, apart from the use of gray literature, as long as they were clinical and/or observational studies. The following steps were carried out: (1) identification of the titles of the records by searching the databases, (2) removal of duplicates, (3) screening and selection of abstracts, (4) assessment of eligibility by means of full-text articles and (5) final inclusion in the study. The studies were selected by two independent reviewers. When the latter did not agree, a third reviewer made the decision whether or not to include the article.

### 6) Data collection process

Data extraction was carried out independently by two researchers, using an electronic form prepared with Microsoft Excel®, and a data compatibility assessment was carried out by a third researcher to evaluate the consistency and clarity of the data. A critical assessment of the quality of the study data was carried out using the Oxford Centre Evidence-Based Medicine Method [18], as well as GRADE recommendations for systematic reviews [19, 20]. The synthesis of the data from this study was based on PRISMA, following the structural logic of IMRD—Introduction, Methods, Results and Discussion [16].

### 7) Risk of bias

Two researchers independently assessed the risk of bias of the included studies. The risk of bias was assessed using the QUADAS-2 tool for systematic reviews of diagnostic accuracy studies. In this tool, the following domains are evaluated for analyzing the risk of bias: 1) patient selection; 2) index test; 3) reference standard; 4) and flow and timing.

### 8) Study selection

**Fig 1:**
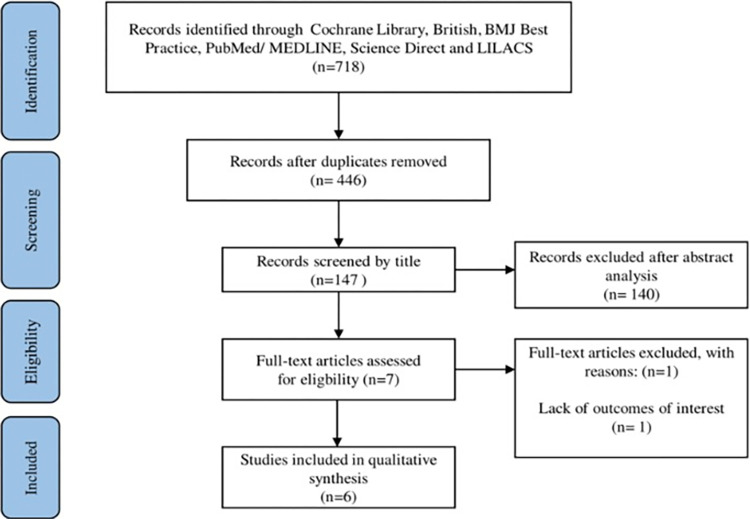
Flowchart for identifying the studies in the systematic review.

**Fig 2:**
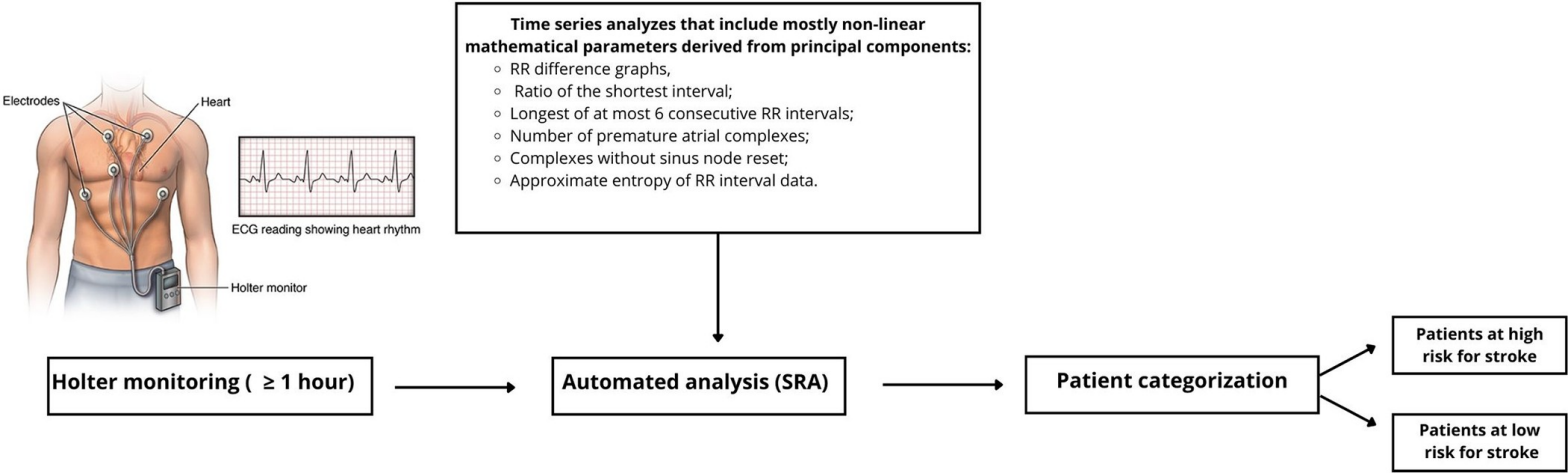
Analysis scheme using the SRA.

Six articles with a total of 2,088 participants met the inclusion criteria. All the studies sought to assess the accuracy of the SRA in predicting the risk of stroke patients presenting with paroxysmal atrial fibrillation in an emergency unit (Fig 1). A diagram demonstrating the realization of the SRA is shown in Fig 2.

## Results

### 1) Individual study results

With regard to the prospective studies, one of these was carried out with 136 stroke patients with no history of atrial fibrillation on admission, a median age of 72 years and an SRA monitoring time of between 1–2 hours were reported. PAF was detected in 29 (21.3%) of the patients using an implantable cardiac device (ICD). The SRA assessed 75 as low risk for PAF, 59 as medium/high risk for PAF and 2 as manifest AF. The SRA had a sensitivity of 72% and specificity of 63% in detecting AF [21] (Table 1).

**Table 1:** Clinical studies on the accuracy of the SRA in predicting the risk of stroke patients having paroxysmal atrial fibrillation.

Study and year of publication	LE	Type	Size	Population	Sensitivity	Specificity	VPP	NPV	Time of SRA
Rizos et al., 2010 [21]	2B	Prospective observational	136	Patients >60 years with acute ischemic stroke or transient ischemic attacks	72%	63%			1–2 h
Reinke et al., 2017 [22]	2B	Prospective observational	105	Patients with cryptogenic stroke	95%	35%	27%	96%	
Uphaus et al., 2017 [23]	2B	Prospective observational	580	Patients with acute ischaemic stroke or transitory ischaemic attack	89.5%	99.3%			
Ross et al., 2018 [24]	2B	Prospective observational	605	Patients with acute stroke	98%	27%	56%	92%	16 h
Adami et al., 2018 [26]	2B	Prospective and retrospective observational	200	Patients with acute ischaemic stroke			38.5%	99.1%	48 h
Gomis et al., 2019 [25]	2B	Prospective observational	462	Patients with acute ischaemic stroke	71%	86%	38%	96%	2 h

In a second prospective single-center study, 105 patients with cryptogenic stroke were reported. A paroxysmal atrial fibrillation detection rate of 18% was observed by MCI (n = 19) (range 6–575 days) and 62% (n = 65) had an increased risk of AF due to SRA. When comparing the predictive accuracy of SRA in relation to MCI, the sensitivity was 95%, the specificity 35%, the positive predictive value (PPV) 27% and the negative predictive value (NPV) 96%. Only one patient with a very low risk predicted by SRA developed AF revealed by ICM after 417 days [22] (Table 1).

A third study, a single-center prospective observational study, involved 580 patients without a history of atrial fibrillation with a mean age of 69.3 ± 12.6 years. It showed that PAF was detected in 19 (3.3%) patients using Holter monitoring and the SRA method assessed 21 patients as being at high risk of PAF (four false-positive results and two false-negative results), leading to a sensitivity of 89.5% and specificity of 99.1% [23] (Table 1).

In another prospective observational study of 605 stroke patients, the median analysis time for SRA monitoring was 16 h (range 2 to 206 h, interquartile range 10 to 36). There were 296 patients with atrial fibrillation, 63 patients with paroxysmal AF and 233 patients with persistent AF. Of the patients with proven AF, SRA categorized 289 as high risk and 7 as low risk (false negative). The overall sensitivity of SRA to detect AF was 98%, the specificity was 27%, the positive predictive value was 56% and the negative predictive value was 92% [24] (Table 1).

More recently, a prospective multicenter observational study was carried out with 462 stroke patients with no history of atrial fibrillation on admission. The median age was 70.0 years, and SRA analysis was performed within the first 2 hours. Among the 462 patients, AF was documented by SRA in 79 (17.1%). Of this group, 45 cases of AF (9.7%) were confirmed after review by an independent, blinded cardiologist. The predictive AF patterns were: sensitivity 71%, specificity 86%, PPV 38% and NPV 96% [25] (Table 1).

Finally, in a retrospective observational study of 200 stroke patients, 99 of whom were retrospective, the mean age of the patients was 71 ± 16 years. The SRA monitoring time was 48 hours. The SRA revealed that 111 patients (56%) were considered to be at low risk of paroxysmal atrial fibrillation, 52 (26%) were at high risk and 37 patients (18%) manifested AF. During the stay in the stroke unit, AF was evident in 58 (29%) patients, 33 (16.5%) as paroxysmal and 25 (12.5%) as persistent. AF was detected in 1/110 low-risk patients (0.9%; 95% CI 0–4.9%), while 20/52 (38.5%; 95% CI 25–52%) high-risk patients were diagnosed with AF. A low-risk SRA level was associated with a reduced probability of subsequent PAF detection (1/111, 0.9%, 95% CI 0–4.3%), while a high-risk SRA level was predictive of an increased probability (20/52, 38.5% (95% CI 25–52%) [26] (Table 1).

### 2) Risk of bias and quality of evidence

The studies were classified as having a very high risk of bias because they were cohort studies with no control group, as shown in Table 2. Two studies did not adopt exclusion criteria for patients with manifest atrial fibrillation on admission [20, 22]. The articles were analyzed for quality and categorized by Grade of Recommendation and Level of Evidence, according to the classification developed by the Oxford Centre for Evidence-Based Medicine. It was found that 100% (n = 6) of the articles were classified as Grade B recommendation—Moderate. All were classified as grade 2 recommendations and level of evidence B (cohort studies), as shown in Table 3.

**Table 2:** Analysis of risk of bias according to the QUADAS-2 too.

Study (author, year)	Patient selection	Index test	Reference standard	Flow and timing
Rizos et al., 2010 [21]	Low	Low	Low	Low
Reinke et al., 2017 [22]	Low	Low	Unclear	Low
Uphaus et al., 2017 [23]	Low	Low	Low	Low
Ross et al., 2018 [24]	Low	High	High	Low
Adami et al., 2018 [26]	Low	Low	Unclear	Low
Gomis et al., 2019 [25]	Low	Low	Low	Low

**Table 3:** GRADE overview.

Quality Assessment						
Studies	Risk of Bias	Inconsistency	Indirectness	Imprecision	Other^a^	Overall Quality of Evidence
**Sensitivity**						
5 studies^a^	High	NA^c^	Serious	NA^c^	None	**Low quality**
**Specificity**						
5 studies^a^	High	NA^c^	Serious	NA^c^	None	**Low quality**
**Positive predictive value (PPV)**						
4 studies ^b^	High	NA^c^	Serious	NA^c^	None	**Low quality**
**Negative predictive value (NPV)**						
4 studies^b^	High	NA^c^	Serious	NA^c^	None	**Low quality**

## Discussion

### Positive/negative predictive value

Of the 6 studies analyzed, 3 showed an analysis of the positive and negative predictive value in relation to the use of SRA. Unanimously, the NPV was above 90%, while the PPV was below 50% in two articles and above in one study. Thus, the method has a high NPV for diagnosing AF.

Gomis et al. analyzed the SRA for 2 hours and found a PPV of 38% and a NPV of 96%. In another study, by Ross et al, the PPV was 56% and the NPV was 96% when the SRA lasted 16 hours. It is therefore worth questioning the length of monitoring, since, when the duration was longer, a higher PPV was obtained, which could alter the diagnostic value of the method.

### Sensitivity and specificity

Of the 6 studies included, 5 analyzed the sensitivity and specificity of SRA. In all of them, sensitivity was higher than 70%, with the highest percentage, 98%, with 16-hour monitoring and the lowest, 71%, with 2-hour monitoring. Although there seems to be a certain relationship between higher sensitivity and a longer monitoring time, all of them showed a high percentage, even with a shorter monitoring time.

With regard to specificity, in three of these studies the specificity was greater than 60%, with monitoring times between 1 and 2 hours. In the other two studies, Reinke et al. and Ross et al. showed a specificity of 35% and 27%, respectively, the latter with 16-hour monitoring. It can therefore be seen that a longer monitoring time did not influence the method’s improved specificity.

### Summary of evidence

This systematic review showed that there is evidence that the Stroke Risk Analysis software has a significant negative predictive value for the detection of paroxysmal atrial fibrillation, despite the quality of the studies being classified as moderate according to GRADE. Thus, it can indicate, albeit in a reserved manner, the contribution of the SRA in the selection of patients at high risk of PAF who will be referred for implantable cardiac monitoring to continue treatment and diagnostic investigation. Another advantage would be a reduction in cost, as patients at low risk of paroxysmal AF would not need longer monitoring by ICM or Holter. The fact that studies have been classified as moderate quality with a grade B recommendation, can be justified by the lack of randomized clinical trials.

The cost-effectiveness of SRA should be compared with the different diagnostic methods developed for AF detection and AF risk prediction. Chang et al. (2022) published a study showing a good correlation between AF detection using commercial smartwatch photoplethysmography recordings compared to that based on Holter tracings (24h-ECG) with a sensitivity of 97.3% [27]. Khurshid et al. (2022) published a study demonstrating the use of a convolutional neural network to infer the 5-year risk of incident AF using 12-lead ECGs from 45,770 patients and compared the predictive accuracy of this technology with the CHARGE-AF clinical risk score with comparable discrimination [28].

The use of portable electrocardiogram (ECG) devices has also been proposed as an alternative for detecting atrial fibrillation. A recent study evaluated the effectiveness of using a portable ECG device in 1,001 participants [29]. The results showed a sensitivity of 79.3% and a specificity of 99.8% in detecting atrial fibrillation. The applicability of this method was also tested by a multicenter study with a sample of 100,000 participants using a portable ECG device, which found a prevalence of atrial fibrillation of 1.04%, suggesting that this technology may be useful in the early identification of the condition in asymptomatic patients [30].

Furthermore, it is important to compare the efficacy of SRA with well-established methods in the health field. A recent study compared the performance of Stroke Risk Analysis (SRA) with the Framingham Stroke Risk Profile (FSRS) in predicting the risk of stroke in an elderly population. The results showed that SRA performed significantly better than FSRS in predicting stroke risk [31].

Pérez-López et al. compared the SRA method with Optical Coherence Tomography (OCT) in the early detection of stroke in patients with atrial fibrillation. The study showed that SRA was more effective in detecting strokes than OCT, with a sensitivity of 93.2% and a specificity of 97.8% compared to OCT, which had a sensitivity of 85.7% and specificity of 91.3% [32].

It is also important to compare the results of SRA with other diagnostic modalities for the early detection of strokes in patients with atrial fibrillation. One study showed that SRA was more effective at predicting stroke than biomarkers, with an area under the curve (AUC) of 0.88 for SRA compared to 0.73 for biomarkers [33].

These studies suggest that SRA may be a more effective tool in the early detection of stroke compared to other methods, such as the FSRS scale, OCT and biomarkers. However, it is important to note that each method may have its strengths and limitations, and it is necessary to assess each patient individually to determine the best approach for early stroke detection.

Although the use of mobile technology and wearable devices to detect atrial fibrillation is promising, it is important to consider its limitations. One is the potential for false positives and false negatives, which can result in unnecessary tests or missed diagnoses, respectively. Another limitation is reliance on patient adherence and engagement, as the accuracy of these devices can be affected by poor adherence to device use and data transmission. Therefore, the use of mobile technology and wearable devices for the detection of atrial fibrillation should be considered as auxiliary tools to support clinical decision-making, rather than stand-alone diagnostic tools.

### ● Limitations and strengths

The strength of the work is the fact that a significant role of the SRA in detecting patients at high risk for FAP was identified, taking into account the analysis of NPV, PPV, sensitivity and specificity of the studies, as well as considering the quality of studies through GRADE.

The assessment of paroxysmal atrial fibrillation in acute stroke is often poorly defined, with no clear distinction between AF and PAF, as well as a failure to report the duration of PAF episodes, which are important points for assessing the results. The statistical analysis of PAF in some studies assessed the accuracy of detecting only patients with PAF, while others assessed those with paroxysmal and permanent AF, a point that also makes it difficult to compare and group data. The variation in electrocardiographic monitoring time between patients in the studies indicates greater detection of AF in patients who had longer monitoring times. Finally, the lack of high-quality randomized studies and the fact that there are only observational studies are important limitations that should be taken into account.

## Conclusions

SRA is reliable in predicting PAF with a high negative predictive value (between 96 and 99.1%). Thus, SRA can be a pre-selection tool with implications for identifying patients at higher risk of paroxysmal AF as a possible cause of stroke who may benefit from ICM monitoring. A combination of SRA and implantable cardiac monitoring is a promising strategy for detecting undiagnosed paroxysmal atrial fibrillation. However, high-quality randomized clinical trials are needed to demonstrate its medical applicability with favorable results compared to currently used diagnostic methods.

## Supporting information

S1 Checklist PRISMA checklist. (DOCX)

S1 Dataset (PDF)
