# Supplementary figures and images for: Is Stroke risk analysis (SRA) a reliable method for predicting atrial fibrillation? A systematic review
Source: PLoS One. 2024 Jun 25;19(6):e0305339. doi: 10.1371/journal.pone.0305339 (PMC11198814; doi:10.1371/journal.pone.0305339)

## Minimal data set

Forest plot of sensitivity and specificity of included studies.

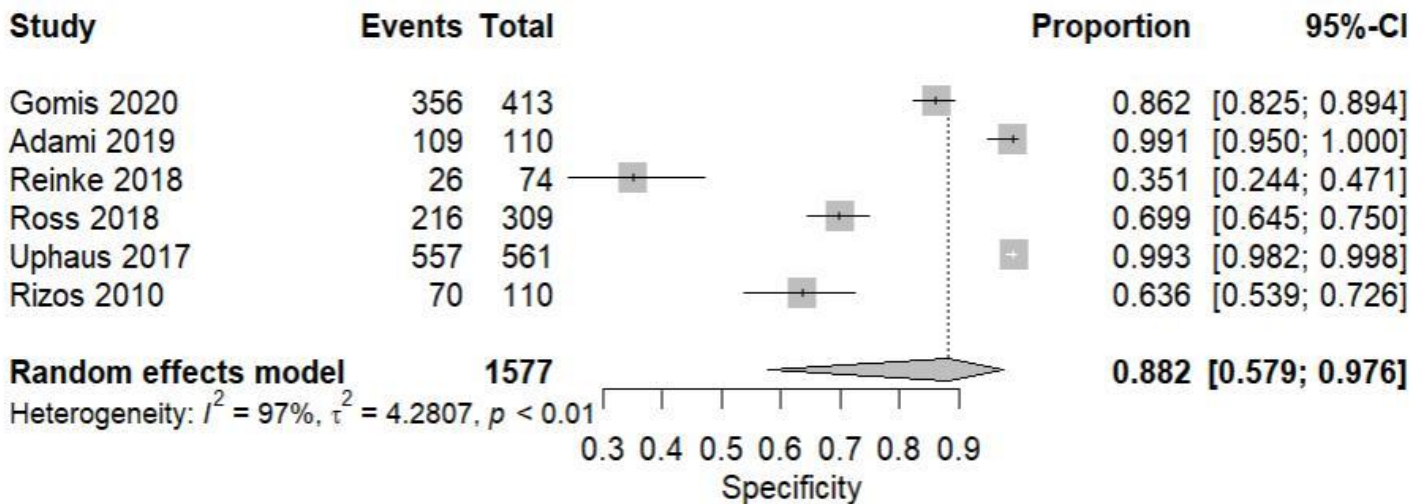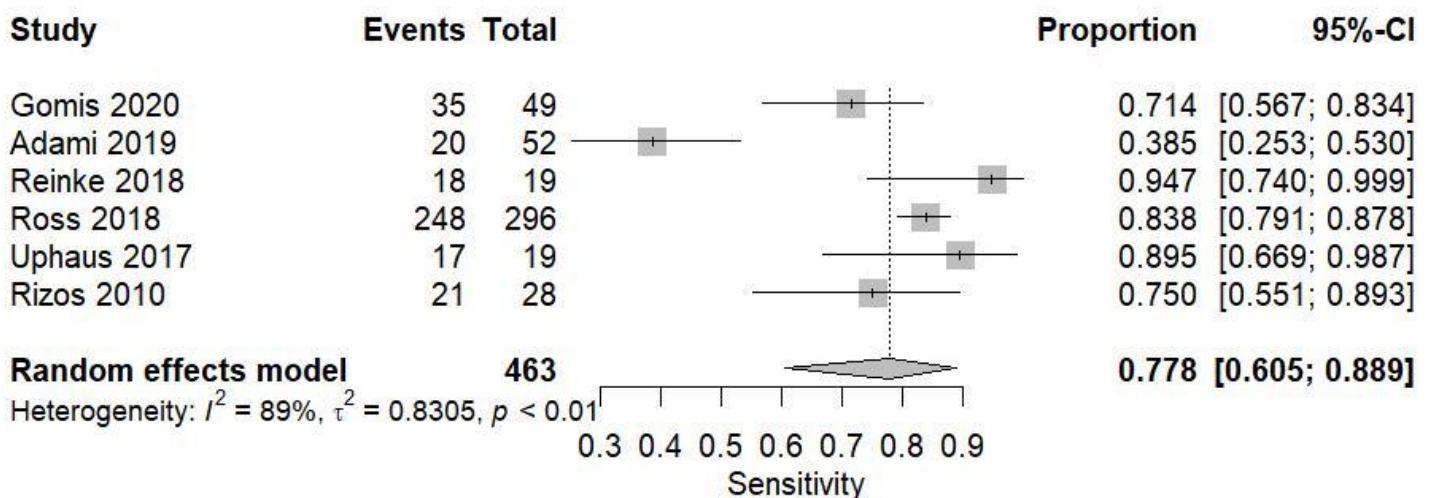

Supplement: S1 Dataset — (PDF) [file pone.0305339.s002.pdf]
